# A literature review of sleep problems and neurodevelopment disorders

**DOI:** 10.3389/fpsyt.2023.1122344

**Published:** 2023-02-23

**Authors:** Abeer Al Lihabi

**Affiliations:** Taibah University, College of Medicine, psychiatry, Al Madinah AL Munawara, Saudi Arabia

**Keywords:** attention-deficit hyperactivity disorder, intellectual disabilities, electroencephalography, autism spectrum disorders, sleep disorder

## Abstract

**Introduction:**

Sleep is an incredibly complex process that goes beyond relaxing and body resting. Disturbance in sleep leads to several short-term and long-term consequences. Neurodevelopmental diseases such as “autism spectrum disorder” (ASDs), Attention-deficit hyperactivity disorder (ADHD), and intellectual disability commonly experience sleep disorders that affect their clinical presentation, daily function, and quality of life.

**Discussion:**

The incidence of sleep problems in ASD patients ranges from 32 to 71.5%, especially insomnia, while an estimated 25–50% of people with ADHD report having sleep issues in clinical settings. The incidence of sleep issues is widespread in persons with intellectual disabilities, reaching up to 86%. This article is a literature review covering the neurodevelopmental disorder interaction with sleep disorder and different management.

**Conclusion:**

Disorders of sleep are key concerns in children with neurodevelopmental disorders. In this group of patients, sleep disorders are common and tend to be chronic. Recognizing and diagnosis of sleep disorders will enhance their function, response to treatment, and quality of life.

## Sleep disorder

Sleeping is an incredibly complex process that goes beyond relaxing and body resting. It is a state of involuntary activity in which the brain is relatively still and responds to internal stimuli during NREM, and 20% is approximately active during REM. The exact purpose of sleep is not fully understood. Sleep has many functions, including neuronal plasticity, memory consolidation, immune function, growth, and mental health. Sympathetic overtone, an increase in the activity of hypothalamic–pituitary–adrenal axis, metabolic disorders, and inflammatory responses are factors that may cause disturbance in the sleep rhythm. Disturbance in sleep leads to several short-term consequences such as emotional disturbance, increase in stress response, mood disorder, cognitive and performance deficits, somatic pain, and reduced quality of life. Sleep disturbance influences teenagers’ mental health, academic performance, and risk-taking behaviors. Sleep disturbance in children is linked to behavioral issues and impaired cognitive performance. In those who are otherwise healthy, sleep disturbance can have long-term effects such as dyslipidemia, hypertension, cardiovascular disease, problems with weight, type 2 diabetes, metabolic syndrome, and colorectal cancer (1–3). The basic form of normal sleep organization is called sleep architecture. The sleep cycle is composed of two phases: non-rapid eye movement (NREM) sleep and rapid eye movement (REM). Phases 1, 2, 3, and 4 of NREM sleep form a continuum of relative depth. Every individual has distinctive characteristics of sleep, such as variations in eye movements, muscle tone, and brain wave patterns. Electroencephalogram (EEG) recordings, which monitor electrical patterns of brain activity, have been used to show sleep cycles and phases (4). The aging process has continuous and considerable effects on sleep architecture. There are noticeable differences between childhood and adulthood in sleep initiation and maintenance, the duration spent in each phase, and overall sleep efficiency. Studying age-related decreases in sleep efficiency is a general trend. Though the effects of insufficient sleep are generally well established, the causes are complicated and poorly understood. Therefore, when examining sleep stages in children of different ages, it is critical to take their unique characteristics into account. However, examining sleep characteristics according to age enables a more in-depth comprehension of how sleep contributes to effective aging and human development. Sleep disorders according to DSM-5 are insomnia, parasomnia, breath-related disorders, hypersomnolence, narcolepsy, circadian rhythm sleep–wake disorder and substance, and medication-related disorder (5).

### Analysis of phenotypes for sleep disorder

There are several types of sleep assessment methods that should be customized for each child. Subjective methods including parent-reported surveys and sleep diaries are among the most frequently employed methods in the analysis of sleep disorders in human studies. They offer various advantages, including non-invasive acquisition and low costs. Children’s Sleep Habits Questionnaire (CSHQ) is considered one of the most popular parent-completed surveys. It is a tool for assessment of sleep in school-aged children based on parental reports (6). Another method is the Electroencephalography (EEG). It involves two electrodes that are attached to the patient’s scalp. It provides a recording of the brain’s electrical activity throughout sleep and weakness (7). A method known as a polysomnogram (PSG) is regarded as the benchmark for the objective assessment of sleep compared to a single-channel EEG (8). It incorporates physiological indications of normal and abnormal brain electrical activity, sleep architecture, sleep stages, and sleep quality, as well as eye movements and physical activities during sleep. Actigraphy provides a non-invasive evaluation of limb activity using an accelerometer to identify episodes of sleep and wakefulness. It enables the collection of data over several days in unstructured settings. The reliability of actigraphy with PSG was examined and revealed a strong relationship between PSG and actigraphy measures (>0.80) for sleep latency, length, and efficiency (9). Similar to actigraphy, videosomnography’s benefits come from its objective documentation over a long period (10). It can also be used to record unusual occurrences like nighttime parasomnias. However, using videosomnography in child sleep research comes with several difficulties.

## Sleep problems and autism spectrum disorders

Autism spectrum disorders (ASDs) have a wide range of clinical symptoms that are connected to social communication and interaction. Restrictive, repetitive, and stereotyped behaviors and interests are common in ASD. They have persistent difficulties in reciprocal social interaction and communication across a variety of circumstances. High levels of co-occurring behavioral difficulties are frequently present in children with ASD. According to the most recent report from the United States, the incidence was 1/54 in 2020. One of the most common features of ASD is the sleep disorder that results from the interaction of several factors such as psychological, biological, family factors, environmental, and child practice methods that might not be sleep-friendly (11).

### Incidence of sleep disorders in patients with ASD

The incidence of sleep problems in ASD patients ranges from 32 to 71.5%. Children and adolescents with ASD are more likely to experience sleep difficulties, especially insomnia, with incidence rates ranging from 40 to 80%. This wide range of incidence may be due to the different sleep problems assessment methods and different criteria such as different cut-off scores (12). After adjusting for family variables including poor child-rearing practices (for example, little parental supervision at bedtime), and noisy, or stressful environments, children with ASD are also more prone to experience sleep disturbances compared with their normal relatives; 47 and 16%, respectively, (13).

### The causes of sleep difficulties in children with ASD

ASD is a multifactorial disease that is affected by multiple factors including neurological, genetic, immunological, and environmental factors. Several neurotransmitters like melatonin, GABA, and serotonin are required to create a regular cycle of sleep and wakefulness. Sleep may be affected by any problems with these neurotransmitters’ synthesis (14). The hormone melatonin aids in synchronizing and preserving the circadian cycle. Autism may have improper melatonin regulation. The integrity of synaptic transmissions and the control of melatonin in ASD may both be influenced by clock genes (15). Melatonin exogenous therapy has been demonstrated to improve sleep schedule in ASD kids. Children with ASD showed decreased activity of the final enzyme in the production of melatonin, indicating lower levels of melatonin. This enzyme is encoded by the N-acetylserotonin O-methyltransferase gene (16). GABA is the neurotransmitter that induces sleep by inhibiting cells that are involved in arousal functions. It is produced from the preoptic area which is the sleep area in the hypothalamus. The Bidirectional Theoretical Framework of Sleep Disturbance provides an overview of the various risk factors that can affect the development of sleep issues in people with ASD (12).

### Effect of sleep disturbances in ASD patients on caregivers

Increased parental sleep problems and maternal stress have been associated with sleep abnormalities in children with ASD. Sleep issues can significantly affect a child’s quality of life, daily functioning, and family dynamics, adding stress to everyone involved. This has also been linked to more challenging behaviors in ASD children during the day, as well as an influence on the ability to control mood. Sleep quality has been linked to common medical disorders such as upper respiratory problems and vision problems. Poor appetite and a decrease in the rate of growth have been linked to increased nighttime awakening and a reduced desire to go to sleep. Sleep disturbance in ASD children has been linked to increased aggression, hyperactivity, and social issues that may be markers of poor mental health outcomes.

### Sleep disorders management

It is crucial to conduct early and frequent screenings for sleep impairment and its related conditions.

#### Non-pharmacological management

Sleep disturbances in children and adolescents with ASD must be managed on both environmental and behavioral levels. Parents must set bedtime routines and provide a relaxing bedroom atmosphere for their children. These environmental and behavioral strategies can improve their sleep, despite the fact that they are challenging to apply (17). The Sleep Committee of the Autism Treatment Network developed the sleep tool kit (STK), which is a customized behavioral modification tool for children and adolescents with insomnia. STK advocates three approaches: visual scheduling of good evening behaviors, a supplemental relaxing module to reduce arousal levels, and a faded bedtime regimen to sleep when tired. Breathing techniques, muscular relaxation exercises, yoga, massage, mindfulness training, and warm baths are additional soothing modules that aid those patients controlling arousal and anxiety. Taking the developmental characteristics of ASD children into consideration, it is reported that positive routines, unmodified and progressive extinction, and overnight fading are more beneficial in children under 5 years, but older children and adolescents benefit more from cognitive-behavioral therapy (CBT) (17).

#### Pharmacological management

Medical treatment is considered if the children do not respond to behavioral therapy.

##### Role of melatonin

It has been suggested that a lack of sociability may be related to sleep difficulties and circadian rhythm disruptions in ASD patients. Indeed, zeitgebers (also known as timeivers) like the natural light–dark cycle, music, and social cues are necessary for the entrainment and synchronization of the circadian clock. Therefore, in ASD patients, poor social cue perception or interpretation may impair the effectiveness of systems that synchronize sleep and wakefulness (18). As an alternative, ASD patients could find it difficult to synchronize with their internal and external settings, leading to eventual rhythm and time problems which affect a variety of fields, including social interaction and circadian cycles. In other words, persons with ASD would experience circadian abnormalities due to their failure to reflect their internal clock on environmental and social rhythms (19).

Melatonin is offered in a variety of over-the-counter preparations ranging from 1 to 10 mg. Most frequently, it is advised to take a dose of 1–3 mg 30–60 min before planned bedtime (46). However, a lower dose (0.5–1 mg) given earlier (3–4 h before night) is advised if a circadian rhythm problem is found. Age or weight has no bearing on the effectiveness of a dose. Melatonin is a pineal hormone that controls the body’s circadian cycle. Melatonin appears to help shorten the time it takes to fall asleep, but its effectiveness at reducing overnight awakenings and other elements of sleep disruptions varies (20). A study included 24 ASD children aged 1–3 years who exhibited improvement in sleep latency as determined by actigraphy when given 1 mg or 3 mg. This treatment improved not just the children’s sleep patterns, but also their conduct and parental stress (21).

##### Antipsychotic medication

This drug class has minimal tolerability and efficacy data for the treatment of insomnia in children. Few trials on the influence on sleep architecture have found that ziprasidone, olanzapine, and risperidone increase slow-wave, although ziprasidone and risperidone reduce REM sleep. Risperidone and olanzapine are two atypical antipsychotic that have been recommended for sleep disorders in children (22). These medications are used for the treatment of insomnia off-label, and it is not advised that they be regularly prescribed for this use, particularly as a first-line pharmacotherapeutic medication. In particular, the Canadian Academy of Child and Adolescent Psychiatry has advised against using them as a first-line line treatment for insomnia in children, adults, or the elderly (23). Other countries have likewise attempted to limit the number of prescriptions that government-subsidized programs may allow.

##### Antidepressant

There is limited evidence on the use and effectiveness of sedative antidepressants, selective serotonin reuptake inhibitors (SSRI), and tricyclic antidepressants (TCA) for the management of sleep disturbances in ASD children. Such medications might be effective if the sleeplessness is accompanied by concomitant psychiatric disorders. Children with comorbid depression may benefit from sedative antidepressants like trazodone and mirtazapine. These antidepressants enhance sleep by reducing the effects of neurotransmitters that promote wakefulness, including acetylcholine, histamine, noradrenaline, and serotonin. As a side effect, the majority of such medications reduce REM sleep and prolong daytime sleepiness. In psychiatric practice, trazodone is widely chosen and employed. Its effectiveness has primarily been shown in people with psychiatric illnesses. Trazodone has a noticeable morning hangover effect due to the antagonism of the 5-HT2A/C and being powerful sedating antidepressant. In contrast, fluoxetine is frequently connected to insomnia. Comparatively speaking to doses used to treat mood disorders, doses used to treat insomnia are typically lower.

##### Alpha-adrenergic agonist

The two main alpha agonists that are frequently used off-label to treat autism-related sleep disorders are clonidine and guanfacine. Clonidine (dosing range: 0.05–0.225 mg/day) significantly reduced sleep initiation and maintenance insomnia in children and adolescents (aged 4–16 years) with autism and neurodevelopmental problems, with good tolerability and few side effects (24). Hypotension, irritability, bradycardia, dry mouth, and REM suppression are some of the side effects of clonidine that may occur, and its rapid withdrawal may result in rebound hypertension and rebound REM (25).

##### Sedative and hypnotics drugs

Hypnotics and sedatives benzodiazepines (BZDs) are routinely given to adults with insomnia. However, because of their side effects, which include drowsiness, headaches, cognitive impairment, dizziness and rebound sleeplessness, and physical and behavioral dependence, they are recommended less frequently to children. Clonazepam was the only benzodiazepine tested for sleep issues in autistic children. Children with developmental disabilities were found to benefit from the treatment of partial arousals, parasomnias, periodic limb movement disorder, and nocturnal biting with clonazepam, an intermediate-acting BZD (26, 27).

##### Other medication

Several medications which are used in the treatment of the Alzheimer’s disease are also found to be effective in the management of ASD symptoms (28). Drugs such as donepezil and rivastigmine are cholinesterase inhibitors that increase the acetylcholine by preventing its destruction. ASD is associated with anomalies in the cholinergic system, according to previously published evidence (29). First, research looking at post-mortem brain samples from people with ASD has discovered cholinergic system anomalies (30, 31).

According to several studies, a large percentage of children with ASD condition experience seizures. It is reported that the incidence of ASD cases that suffered from epilepsy may range from 5 to 38% which is much higher than the incidence of the epilepsy in the normal children population which is 1–2% (32, 33). There is very limited evidence about the use of anti-epileptic drugs in ASD patients. A randomized controlled trial has been valproate in ASD cases (34). They found that valproate monotherapy reduced the irritability and repetitive behaviors in ASD cases (35).

## Sleep disorder and ADHD

One of the most frequently identified illnesses in both children and adults is attention-deficit/hyperactivity disorder (ADHD). It affects 2.9% of adults and 3 to 5% of children. It continues into adolescence and adulthood. The diagnostic criteria of ADHD include symptoms of inattention or/and impulsivity that appear prior to 12 years old, and hyperactivity. Untreated ADHD patients suffer from a decrease in several critical functional domains, including the academic, social, and occupational realms (36).

### Types of sleep disorders

An estimated 25–50% of people with ADHD report having sleep issues in clinical settings (37). Besides, adults who do not have enough time of sleep are more prone to have symptoms of ADHD (38). Such individuals’ sleep disruptions have been linked to concomitant primary sleep problems and/or changes brought on by ADHD drugs (6). Researchers have looked into the connections between ADHD and narcolepsy, insomnia, circadian rhythm sleep disorders (CRSDs), restless leg syndrome, and sleep-disordered breathing (SDB) (39, 40).

### Obstructive sleep apnea and ADHD

Obstructive sleep apnea (OSA) is characterized by partial or total obstruction of the upper airway, which results in interrupted sleep, while SDB is associated with unpredictable breathing rhythm during sleep (41, 42). People with ADHD have a higher incidence of SDB, and those with a history of snoring or possible OSA throughout childhood are associated with a two-fold higher susceptibility of diagnoses with ADHD (43). Through several processes, involving negative effects of hypoxic outcomes, the inflammation that leads to brain, and/or recurrent arousal-based sleep disturbances, SDB influences psychological outcomes. These pathways may change the prefrontal cortex’s neurochemical substrates, resulting in the neurobehavioral abnormalities that underlie the symptoms of ADHD (44).

### Restless leg syndrome and ADHD

Restless leg syndrome (RLS) is a common sensorimotor condition characterized by an intense need to move the legs, which is frequently accompanied by unpleasant leg or (less frequently) body-part feelings. These feelings are particularly uncomfortable in the evening or at night and get better with activity. Due to their need to walk about and the stiffness in their legs, patients frequently have sleeplessness. This comorbidity is thought to be caused by iron deficiency and dopaminergic disorders (45, 46). Even though the incidence of RLS in children is unknown, the disorder affects 10% of adults in the United States. According to the data, up to 44% of people with ADHD have RLS or symptoms similar to it, while up to 26% of those with RLS have symptoms similar to it (47, 48).

### Circadian rhythm sleep disorder and ADHD

The timing of when a person sleeps and is awake is a concern in CRSDs. They result from changes to the circadian clock, its entrainment processes, or a misalignment of the internal circadian rhythm with the external environment. When a person routinely falls asleep and awakens more than 2 h later than is deemed normal, this condition is known as delayed sleep phase syndrome (DSPS). Changes in these processes, reductions in pineal gland volume, and/or anomalies in clock genes have all been discovered in people with ADHD. In adolescents and adults with ADHD, late chronotype and DSPS are typically co-occurring disorders. CRSD and ADHD may share a biological and behavioral etiology (49, 50). Impulsivity control issues might impair a person’s capacity to calm down, causing resistance to going to bed and a delayed start to sleep. It is also suggested that those with ADHD might have a greater circadian preference for the evening and a potential endogenous melatonin rise delay (50).

### Narcolepsy and ADHD

A persistent neurological condition called narcolepsy causes problems with sustaining constant wakefulness and sleep. A diagnosis of narcolepsy needs symptoms of rapid eye movement (REM), sleep dissociation (such as sleep paralysis, hypnagogic/hypnopompic hallucinations, and cataplexy), and disturbed nighttime sleep, regardless of how the clinical presentation manifests itself. In the past, it was discovered that adults with narcolepsy had a twice as high probability of receiving an ADHD diagnosis as children as compared to controls (51). Additionally, data points to children with ADHD experiencing hypo arousal and hypo arousal-related hyperactivity/impulsivity as possible signs of exhaustion (52). Although the relationship between the two disorders is unclear, it has been postulated that EDS in narcoleptics may cause inattention, deficient executive function, and issues with impulse control that are similar to ADHD and react well to psychiatric drugs (53–55). Finally, the overlap of ADHD and narcolepsy symptoms may result in diagnostic ambiguity or incorrect diagnosis of the diseases. Another theory is that the connection could be due to a common pathology in the brain (56).

### ADHD medication

#### Stimulants

The effects of stimulants on sleep vary from patient to patient in those with ADHD, reflecting the intricacy of the relationships between sleep disturbance and ADHD (57). Clinical experience suggests that stimulants generate paradoxical effects, whereby symptom relief can relax patients and encourage sleep, although there is evidence linking stimulants to disturbed sleep in ADHD cases (58, 59). Furthermore, increasing the dosage of a short-acting inducer or using a formula with prolonged action may minimize sleep disruptions caused by an increase in hyperactivity or behavioral disorders at bedtime due to the risk of symptom rebound when the concentrations of the drug in the blood is decreased (60, 61).

#### Non-stimulants

The most frequent side effect associated with atomoxetine that is connected to sleep, in contrast to stimulants, is somnolence (a noradrenaline reuptake inhibitor permitted for the management of ADHD). In atomoxetine placebo-controlled trials, somnolence was observed to present in 15–17% of patients as reported by a 2009 comprehensive review (62). Atomoxetine was found to have less of an impact on subjective sleep measures than methylphenidate and was taken three times per day in a randomized, double-blind trial.

### Management

After evaluation and diagnosis, the first stage of treatment will be psychoeducation. In addition to learning about the prognosis, course, therapy, and probable functional implications of the sleep disorder, the affected individuals and their social entourage will require proper psychoeducation on ADHD symptoms and sleep problems. Additionally, educating people about healthy sleep habits and sleeping patterns will enable non-pharmacological sleep enhancement. It is common practice to use medicine to address sleep disturbances. The choice of medication can be directed to address related issues such as daytime malfunction and should be combined with behavioral techniques. Surgery to remove the tonsils or adenoids is the first line of treatment for children with ADHD and SDB, whereas oral appliances, positive airway pressure devices, or surgery are suggested treatments for adults with OSA and ADHD (63). The sleep environment may need to be changed for people with RLS and ADHD, and behavioral therapies such as iron supplements (64) (for example, ferrous sulfate) or gabapentin (65) may also be investigated, especially for a younger population. In an adults, using dopaminergic substances such as L-DOPA, ropinirole, and pramipexole, in addition to, a recently developed drug called rotigotine may also be a possibility (66).

Treatment options for people with DSPS and ADHD include scheduled melatonin therapy, light therapy (67), and chronotherapy (68, 69). Furthermore, because treatment for DSPS differs from that for insomnia, a clear distinction between the two must be made. Treatment for ADHD and insomnia can differ depending on the age group.

## People with intellectual disabilities and sleep disorders

The incidence of sleep issues in children ranges from 24 to 86%, and they are widespread in persons with intellectual disabilities (70). Adults with mental disabilities are reported to have an incidence of sleep disorders ranging from 8.5 to 34.1%, with a serious sleep problem rate of 9.2%. In one study, it was discovered that 551 older persons with intellectual disabilities had sleep issues in 72% of the cases (47). The treatment of physical and mental health issues in people with intellectual disabilities is an area that needs more study. Studies conducted on people without intellectual disabilities were often the basis for the development of diagnostic and management techniques. The same pattern is observed in persons with intellectual impairments who have sleep difficulties. There is a lack of information specifically on the causes, effects, and treatments of sleep problems in people with developmental disabilities. Assessment and treatment of sleep issues in persons with intellectual impairments can be informed by knowledge of the several types of sleep issues that these people encounter and the numerous factors that affect their sleep (71).

Adults with intellectual disabilities have a higher risk to have sleep issues, which could be due to several factors. In individuals with intellectual impairments, a systematic review of the published literature on sleep problems found links between sleep and several characteristics, such as respiratory diseases, psychoactive drugs, mental health illnesses, and challenging behavior (47). Understanding and taking into account the social, psychological, and biological aspects influencing the higher occurrence of sleep issues in persons with intellectual impairments is crucial for providing person-centered and individualized care. We have looked at several significant contributing elements that must be taken into account when evaluating sleep issues in persons with intellectual disabilities. The association between sleep disturbances and neurodevelopmental diseases like ADHD and ASD has been thoroughly studied above (47).

### Genetic conditions

Our comprehension of the underlying genetic causes of intellectual impairments has recently improved (72). For example, Down’s syndrome is characterized by obesity, hypotonia, and craniofacial anomalies, all of which raise the likelihood of sleep disorders including obstructive sleep apnea (OSA). Similarly to this, those who have cri du chat syndrome have a higher risk of getting OSA (73).

### Environmental and psychological factors

People with mental disorders frequently experience sleep disturbance as the first sign of a decline in their mental health, and low-quality sleep represents a common feature of many psychotic and affective diseases (11). When compared to individuals without cognitive disabilities, those with cognitive disabilities have a higher incidence of mental problems, which helps explain why sleep disturbances are so common in this population (12). When determining the etiology of sleep issues in a person with cognitive disabilities, it is crucial to take the environment’s role in the development of sleep disorders into account. According to Kerr and Wilkinson (13), staffed residential homes may not be the best places to sleep because people may check on residents at odd hours, which would result in more noise and lights that would be disruptive to sleep (13).

### Diagnosis of sleep disorders

Adults with intellectual impairments are frequently given subjective sleep information by their caregivers, who may disagree with the severity of the problem or may even accept sleep disturbance as a symptom of the person’s underlying condition (74).

Because of this, sleep problems are more likely to be noticed by a physician when they induce nocturnal or daytime malfunction, including behavioral disorders, impairing a person’s subjective impression of their quality of life (75). When evaluating sleep disturbances, general population guidelines stress the significance of checking for coexisting medical diseases. This is may be even more crucial for people with intellectual impairments since they are more prone to experience physical health illness that impacts their sleep, such as OSA or epilepsy (76). For example, it is advised that everyone with Down’s syndrome be evaluated for OSA due to the condition’s high incidence in people with Down’s syndrome. When sleep–wake duration (including naps) is irregular or unpredictable, caregiver-completed sleep diaries and/or actigraphy, ideally conducted for at least 2 weeks, can be used. Individuals with intellectual impairments can be evaluated for physical sleep disorders such as OSA and nocturnal epilepsy using home or in-patient sleep examinations (such as pulse oximetry or the gold standard, polysomnography) (77). Even though these tests should always be provided when clinically indicated, if a patient is unable to endure the sleep tests, a practical therapy trial may be necessary. The variety of underlying causes of intellectual disability and the characteristics of related comorbidities make managing sleep disturbances challenging. While intellectual disability psychiatrists can easily address some problems, others will need the assistance of sleep physician and/or primary care givers. According to a study, continuous positive airway pressure therapy can significantly enhance behavior, cognitive function, and subjective drowsiness in individuals with Down’s syndrome and OSA (78). But as this study correctly notes, access to care may be difficult, and as was already said, some individuals with intellectual disabilities might find it difficult to tolerate these tests and treatments. Working together, sleep specialists and psychiatrists may be able to address these issues. For instance, sleep clinics assist the training of mental health nurses for people with intellectual disabilities in exposure treatment to aid those individuals become used to positive airway pressure masks. The initial management for persistent insomnia in the general population is multicomponent cognitive-behavioral therapy (CBT-I), and there are elements of this that can be helpful for individuals with intellectual impairments (74). Understanding the impacts of environment and lifestyle on sleep, such as caffeine use, exercise, and regular sleep schedules, along with lighting, noise, and temperature, can be achieved through education on sleep hygiene (75).

Adults with cognitive disabilities are more prone than the general population to lack appropriate daily activity and regular exposure to natural light, thus even small changes to everyday routine and the sleeping environment can be beneficial (75). The needs of the person should be taken into consideration when making such recommendations, such as lowering external noise for autistic people sensitive to sounds. The evidence is not strong enough to support the use of pharmaceuticals to treat sleep disturbances in adults with intellectual impairments. Melatonin is the drug that has drawn the most attention, maybe due to its favorable side-effect profile and the fact that several trials have demonstrated its efficacy (75). According to a meta-analysis, melatonin consumption enhances total sleep time and reduces the number of wake-ups per night in people with intellectual disabilities. Currently, the pharmacological management of illnesses other than insomnia tends to use the same routes as those for the general public (75).

## Conclusion

Disorders of sleep are key concerns in children with neurodevelopmental disorders. In this group of patients, sleep disorders are common and tend to chronicity. Various solutions are required based on the neurodevelopmental problem, but all patients should get behavioral intervention. Understanding the distinctive characteristics of sleep disturbances in patients with neurodevelopmental disorders is critical for effective therapy.

## Author contributions

The author confirms being the sole contributor of this work and has approved it for publication.

## Conflict of interest

The author declares that the research was conducted in the absence of any commercial or financial relationships that could be construed as a potential conflict of interest.

## Publisher’s note

All claims expressed in this article are solely those of the authors and do not necessarily represent those of their affiliated organizations, or those of the publisher, the editors and the reviewers. Any product that may be evaluated in this article, or claim that may be made by its manufacturer, is not guaranteed or endorsed by the publisher.
